# Caustic ingestion leads to pneumonectomy and right colonic interposition, a unique case report

**DOI:** 10.1016/j.ijscr.2023.108479

**Published:** 2023-07-07

**Authors:** Kenana Tawashi, Yamama Tawashi, Hajar Odah Bashi, Fawaz Al Sharif, Hussam Dalati

**Affiliations:** a Faculty of Medicine, Hama University, Hama, Syria; b University Pediatrics' Hospital, Damascus, Syria

**Keywords:** Caustic ingestion, Esophageal replacement, Pneumonectomy, Colonic interposition, Case report

## Abstract

**Introduction:**

Corrosive ingestion forms serious problem, with various outcomes depending on the time of diagnosis and treatment. We report here a case with rare and dangerous complications.

**Presentation of case:**

A two-year-old girl came to our hospital, complaining of solids' dysphagia. Retrieving her medical history showed that she had ingested a corrosive liquid accidentally. Without knowing the nature of the ingested agent, the local doctor removed it, using nasal gastric tube. This procedure induced vomiting, which in turn led to more damage. She stayed in the area's hospital for 40 days with just supportive treatment. The radiological investigations suggested severe stenosis. The dilation was done, but the patient did not respond after three months of treatment. Therefore, a gastrostomy was done. The esophagus replacement was inevitable, but the parents refused the surgical approach. Three months later, she returned to our hospital complaining of a productive cough. The radiological investigations suggested destruction in the left lung with a high suspicion of tracheoesophageal fistula. The treatment was through a surgical approach by removing the damaged lung tissue and closing the tracheoesophageal fistula. The patient got better after a month of the surgery, which allowed us to replace the esophagus and close the tracheoesophageal fistula.

**Discussion:**

corrosive treatment varies a lot, depending on the patient's situation. Accurate treatment prevents severe and unexpected complications.

**Conclusion:**

More stringent instructions should be enacted among medical providers, corrosive agents' producers, and the public to be more careful when dealing with corrosive chemicals.

## Highlights

• Caustic ingestion can cause unexpected complications in case of late treatment.
• Esophageal replacement should be done to avoid more complications.
• More stringent instructions should be enacted among medical providers, and the public to be more careful when dealing with these chemicals and products.

## 1 Introduction

Caustic ingestion leads to a chemical reaction between this substance and the tissue. Caustic agents are composed of acids and bases depending on their chemical nature [1]. Acidic agents damage tissues through the coagulation necrosis process. Whereas, basic agents work through liquefaction necrosis. In addition, basic agents are colorless, tasteless, and odorless, which facilitates ingesting much more amounts of them [2,3]. The most affected organs are the esophagus and stomach [2]. Many factors determine the severity of the injury including; the substance's concentration, the ingested amount, the agent's type and pH, and the contact period [2,4]. Caustic agents have been usually ingested by two major groups; children, who often consume small amounts accidentally, and adolescents and young adults, who tend to ingest corrosive agents in suicidal attempts [2]. In this paper, we discussed a poorly treated case, where we have serious unexpected complications, which could have been avoided by accurate diagnosis and treatment. This work has been reported in line with the SCARE criteria [5].

## 2 Presentation of case

**Fig. 1:**
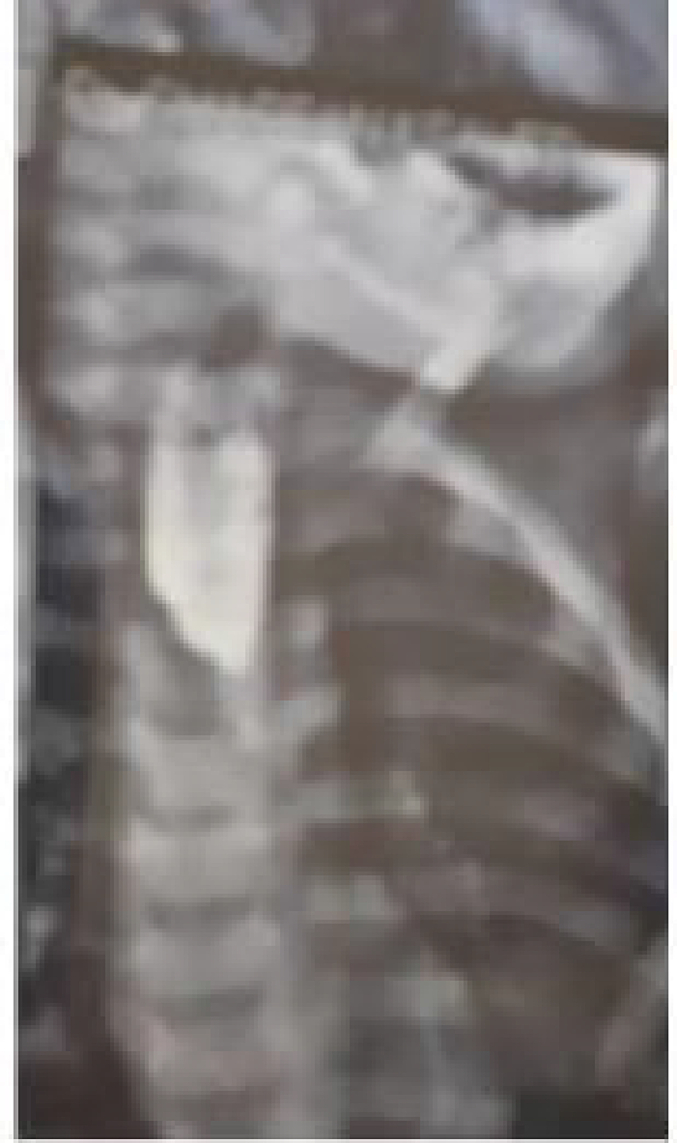
Esophagus and stomach Barium X-Ray in the first visit to hospital shows severe stenosis (the barium gathered in the lower part of the esophagus).

**Fig. 2:**
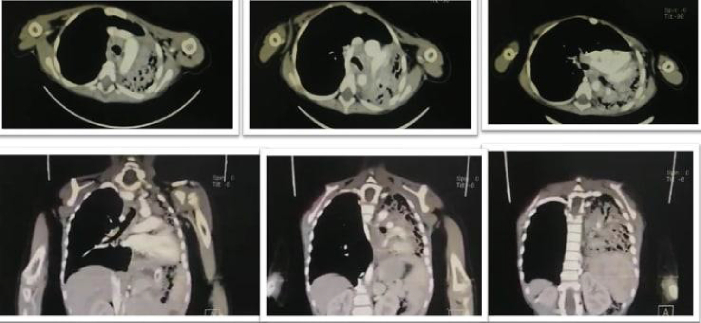
The CT scan images show atelectasis in the left lung with the destruction of the lung tissue (shows wide infiltrations /alveolar pattern/ in the left lung).

**Fig. 3:**
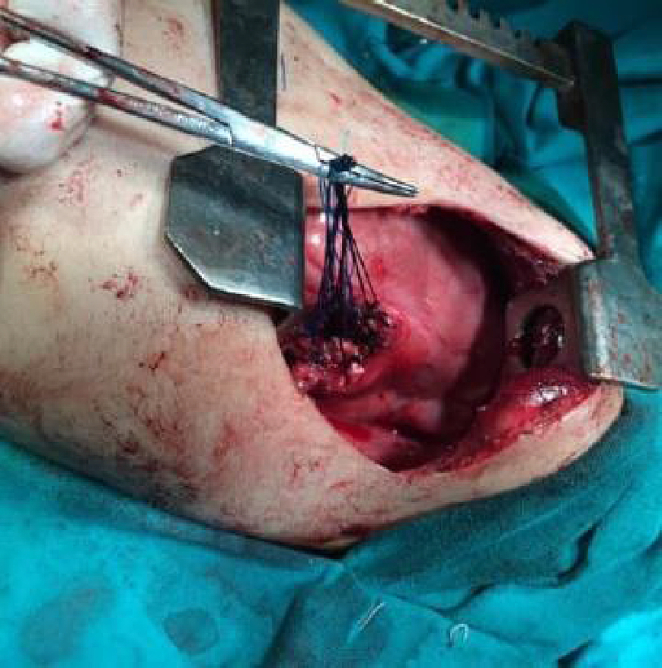
Image from the surgical approach (Pneumonectomy).

**Fig. 4:**
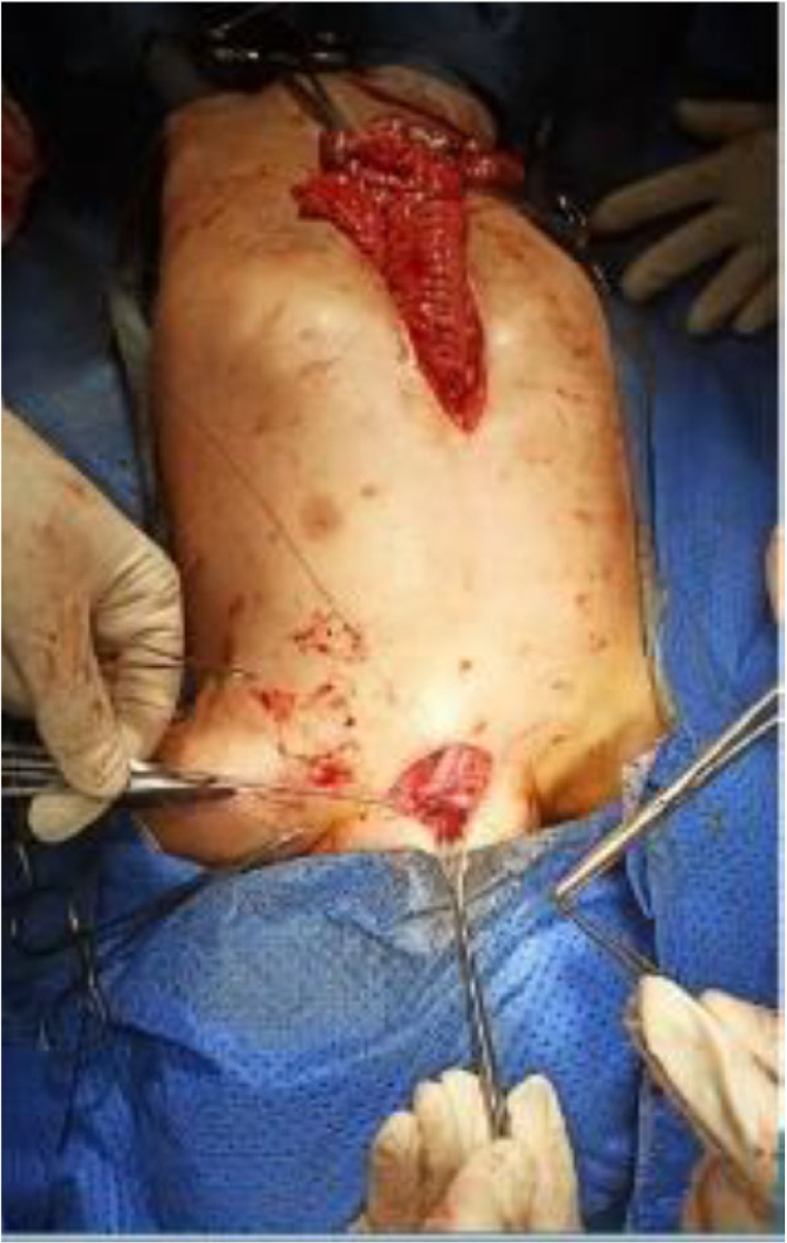
Images from the surgery show the colonic conduit and the anastomoses, also shows a mid-incision above the umbilical and a cervical incision above the lateral side of the manubrium where we found a tracheoesophageal fistula.

**Fig. 5:**
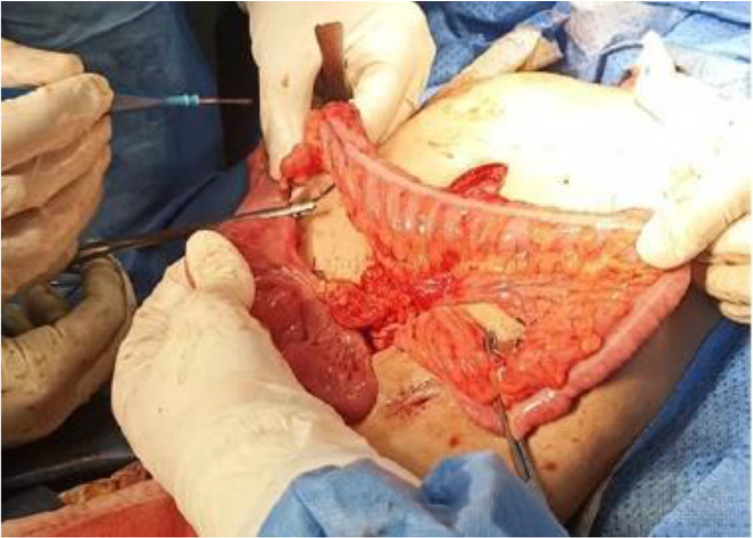
Images from the surgery show the colonic conduit and the anastomoses, also shows a mid-incision above the umbilical and a cervical incision above the lateral side of the manubrium where we found a tracheoesophageal fistula.

A two-year-old girl came to our hospital, complaining of solids' dysphagia. She was pale and thin. Her physical examination was normal. Retrieving her medical history showed that she had ingested a corrosive liquid (NaOH) accidentally 50 days ago. After 5 min of ingestion, she developed nausea, vomiting, hypersalivation, and redness. Without knowing the ingested agent, the local doctor removed it by inserting a nasal gastric tube (NGT). This procedure induced vomiting, which in turn led to more damage. The patient spent 40 days in the local hospital, and the treatment was limited to intravenous solutions, antibiotics, and feeding by NGT because of the lack of resources. Expect that, the history of the patient was uneventful. The chest X-Ray was normal. The upper endoscopy and barium X-Ray showed severe stenosis (Fig. 1). The laboratory investigations showed the following values (Table 1). The dilation was done using Savari dilators, but the patient did not respond after three months of treatment. Therefore, the gastrostomy was done during this period to feed the patient and the esophagus replacement was inevitable. The parents refused the surgical approach and the patient was discharged. Three months later, she returned to our hospital complaining of a productive cough. The physical examination showed a conscious, berserk, pale, and fatigued girl with fingers clubbing. The general appearance revealed signs of malnutrition and dyspnea. The vital signs were presented in Table 2. Examining the respiratory system revealed an absence of respiratory sounds with dullness on the left side and crackles and wheezing with tympanicity on the right side. The chest X-Ray showed wide infiltrations (alveolar pattern) in the left lung, closed left costophrenic angle, shifting of mediastinum to the left, and hyperventilation in the right lung. A computed tomography scan (CT) showed atelectasis in the destructed left lung (Fig. 2). These radiological findings indicated a tracheoesophageal fistula. We removed the left lung after finding it non-functional. We tried to isolate the esophagus and remove it, but because of the intense adhesions and inflammation, we could only isolate the distal part of it without finding any fistula within it (Fig. 3). The patient got better after a month of the surgery, which allowed us to replace the esophagus. The surgery started with a mid-incision above the umbilical and a cervical incision above the lateral side of the manubrium where we found a tracheoesophageal fistula. Because of the severe adhesions, we excised as much as we can and then closed the fistula. After an appendectomy, we chose an appropriate conduit from the ascending and transverse colon with its vessels and used the retrosternal route to establish the alternative conduit (Fig. 4, Fig. 5). The patient stayed at the intensive care unit for three days after the surgery and depended during this time on mechanical ventilation. The general situation of the patient got better and she was discharged from the hospital after a month.

**Table 1:** It shows the laboratory values during the first visit to our hospital.

WBCs	Neutro	Lymph	HB	MCV	PLT	PTT	Fe
9.6*10^9^ /L	50 %	42 %	8.6 g/dl	fl 56	403,000 /L	27 s	32 μg/dL
CRP	ALB	TP	Urea	Creat	SGpt	Gluc	Zn
5.5 mg/dl	3.9 g/l	6.2 g/dl	11 mg/dl	0.56 mg/dl	46 U/L	99 mg/dl	7 μmol/L

**Table 2:** It shows the vital signs during the second visit to our hospital.

Temperature	Blood pressure	Respiratory rate
39 °C	90/45 mmHg	40/min


Heart rate	Glasgow scale	The oxygen saturation in the room's air
140 pets/minute	15/15	89 %

## 3 Discussion

Corrosive ingestion is a serious problem, affecting many groups of people. Children from low-income countries are the most affected group [6]. The responsible factors include physiological cases, low educational level, young maternal age, lack of attention, and rural areas. The clinical presentation varies a lot, half to two-thirds of children are usually asymptomatic (in some cases there are not any symptoms or complications and in others symptoms take time to appear). The symptoms and signs often involve the respiratory system, gastrointestinal system, and ear-nose-throat system [7]. Many indicators help us in estimating the severity of injury such as the category of the substance, its concentration, amount, and timing [1]. Laboratory tests contribute in evaluating the general situation like CBC, serum electrolyte, pH, serum lactate, and β-HCG in young women [8]. Radiological investigations that include chest X-ray, ultrasound imaging, water-soluble contrast swallow and meal, CT, magnetic resonance imaging (MRI), and endoscopy, are core stones in such cases. Erect chest X-Ray can reveal mediastinal and subdiaphragmatic air. Intraluminal ultrasound is used to estimate the depth of esophageal injury. Contrast swallow helps in diagnosing esophageal perforation and strictures formed later [2,7]. CT scan determines the spread and depth of the injuries and tissues' perforation [2,3]. Esophagogastroduodenoscopy, which is the primary method in determining the grade of the injury, is recommended to be performed during the first 12 to 48 h of the ingestion. Endoscopy is contraindicated in hemodynamic instability, respiratory distress, and suspected perforation. Zargar's classification is used to estimate the degree of injury by endoscopy (Table 3). Some studies connected the grades of injury with the outcomes [2,9]. The complications of caustic ingestion vary a lot [10]. Esophageal perforation is a dangerous and life-threatening complication that can happen at any time during the first 2 to 3 weeks of ingestion. Esophageal strictures are the most common chronic complication of corrosive substance ingestion. It happens in 70 % of patients with grade IIB and more than 90 % of patients suffer from grade III [2,3]. Esophago-respiratory fistula is a rare complication that happens in 0.3–0.5 % of cases, the related symptoms are coughing after swallowing, dysphagia, belching, aspiration, and pneumonia [3]. Esophageal cancer as a long-term complication is a serious problem that happens in 1–2 % of patients [2,3,7]. Our patient underwent a Pneumonectomy as a complication of caustic ingestion. This dangerous and unexpected sequel happened over two years. Caustic ingestion treatment should start at the time of ingestion by removing and identifying the ingested agent, drinking neutral liquids, and avoiding inducing vomiting. The intravenous maintenance fluids and NGT are necessary in some cases to keep the body balanced while feeding through the oral route should be avoided. Using of antibiotics and steroids is still disputed [7]. Parenteral analgesics are prescribed for severe pain [1]. In the case of transmural necrosis, emergency surgery is the choice to avoid perforation, peritonitis, mediastinitis, and death [8]. Some studies encourage several procedures to prevent stricture formation like steroids, nasogastric tube placement, oral nystatin suspension, and proton pump inhibitors [7]. However, the formed strictures are treated by dilation, stents, and surgery. Delaying initiation of dilation is associated with an increased likelihood of esophageal replacement (as in our case). This procedure was achieved with Bougies and balloon dilators [2,7]. Esophageal replacement, which is essential in 5.7 % of caustic strictures, is the terminal solution for formed strictures [7]. The indications for replacement are long gap esophageal atresia, peptic or caustic strictures, anastomotic strictures, and other rare disorders. The used conduit are gastric transposition, gastric tube, colon interposition, and jejunal interposition [11]. The used routes to establish the conduit are transpleural, retrosternal, and posterior mediastinum. The retrosternal route is an easy choice and could be used when other options are not accessible due to inflammation and previous surgeries. Its disadvantages include the long route, angulation of conduit, and difficulty of cardiac surgery later. [12]. In our case, we use colon interposition. The most commonly used part is the left transverse colonic graft; ascending or descending colon can also be used [11]. The selection of the appropriate part depends on the required length and the colonic vascular anatomy [13]. This conduit has many advantages such as adequate length, acid resistance, and good blood supply. We used the retrosternal route in our case because of the presence of inflammation and fibrotic tissues around the esophagus, so the posterior mediastinal route was contraindicated. Using this route needs a long conduit so we used ascending and transverse colon, and avoided the use of stomach. The stomach was avoided also because of the previous gastrostomy and the associated gastroesophageal reflex and pulmonary complication that followed (our patient had previously had Pneumonectomy) [13].

**Table 3:** Zarger classification.

Zargar classification	Description
0	No evidence of injury
1	Erythema and edema
2A	Bleeding, erosions, blisters, superficial ulcerations
2B	Deep lesions
3A	Focal deep ulcerations with necrosis
3B	Extensive deep ulcerations with necrosis
4	Perforation

## 4 Conclusion

This paper reports a case of caustic ingestion, with unexpected and dangerous complications. Such complications highlight the importance of rapid and accurate treatment and encourage people to be more aware of the life-threatening consequences of poor treatment. More stringent instructions should be enacted among medical providers, corrosive agents' producers, and the public to be more careful when dealing with these chemicals and products.

## Funding

None.

## Ethical approval

This case report is exempt from ethical approval because it does not have any personal information about the patient inside it.

## Consent for publication

Written informed consent was obtained from the patient for publication of this case report and accompanying images. A copy of the written consent is available for review by the editor-in-chief of this journal on request.

## Declaration of competing interest

None of the authors has any conflict of interest to disclose. We confirm that we have read the journal's position on issues involved in ethical publication and affirm that this report is consistent with those guidelines.
